# Slowly expanding lesions are a marker of progressive MS – No

**DOI:** 10.1177/13524585211017020

**Published:** 2021-09-03

**Authors:** Douglas L Arnold, Shibeshih Belachew, Arie R Gafson, Laura Gaetano, Corrado Bernasconi, Colm Elliott

**Affiliations:** NeuroRx Research, Montreal, QC, Canada/McGill University, Montreal, QC, Canada; Biogen Digital Health, Biogen, Cambridge, MA, USA; Biogen Digital Health, Biogen, Cambridge, MA, USA; F. Hoffmann-La Roche Ltd, Basel, Switzerland; F. Hoffmann-La Roche Ltd, Basel, Switzerland; NeuroRx Research, Montreal, QC, Canada

In recent years, highly effective therapies have been developed that can essentially stop the formation of new white matter (WM) lesions in multiple sclerosis (MS). Unfortunately, the efficacy of such therapy to prevent the formation of new lesions visible on magnetic resonance imaging (MRI) has not been associated with comparable efficacy in preventing the development of confirmed disability progression. For example, ocrelizumab suppresses new or enlarging lesion formation by >90% but reduces confirmed disability progression by only 25%.^1^

The reason for this disparity, like the cause(s) of the progressive disability itself, remains unclear; one candidate is chronic inflammation that continues to damage myelin and axons within pre-existing lesions, variably referred to as chronic active lesions, mixed active/inactive lesions, smouldering lesions or slowly expanding lesions (SELs). The characterization of histologically defined chronic active lesions as slowly expanding is based on the assumption that the observed rim of activated microglia was associated with expansion of the lesion during life. We will use the term chronic active lesions to refer to lesions defined histologically to distinguish them from SELs defined on MRI.

## MRI detection of chronic active lesions

Changes over time within existing MS lesions are poorly characterized on conventional MRI where they are typically identified as foci of hyperintensity on T2-weighted images. This binary classification is indifferent to any ongoing pathological changes within the lesions and special techniques are required to detect chronic activity in vivo.

## Phase-rim lesions

One approach is based on the detection of iron associated with activated microglia at the periphery of some of these lesions. This is done using susceptibility-weighted imaging to identify lesions with paramagnetic phase rims. Such lesions, which are referred to as phase-rim lesions (PRLs) or iron-rim lesions probably correspond to the 40% or so of chronic active lesions that are associated with iron accumulation.^2^ PRLs have been found in all forms of MS where they have been looked for (progressive multiple sclerosis (PMS), relapsing multiple sclerosis (RMS), and even radiologically isolated syndrome).

## SELs

A second approach is based on the detection of SELs directly on conventional MRI scans.^3^ This approach uses calculated deformation fields to detect foci of gradual and concentric expansion within existing T2-lesions on serial MRI scans.

It is important to appreciate that this approach to the detection of lesion expansion is fundamentally different from that used for the detection of so-called ‘enlarging lesions’, commonly reported in counts of ‘new or enlarging T2 lesions’ in clinical trials. Methods for the detection of enlarging lesions in the context of ‘new or enlarging lesion counts’ (which vary from laboratory to laboratory) have been designed to detect what are essentially new foci of acute activity that are connected to areas of existing T2-signal abnormality and therefore do not qualify as ‘de novo’ new lesions (which by definition have to be surrounded by normal-appearing WM).

MRI has been used to detect SELs in a large number of people with MS representing different populations.

Elliott et al.^3^ have reported SELs in 1344 RMS and 555 primary progressive multiple sclerosis (PPMS) subjects from the pooled populations of the two identical phase III, multicenter, randomized, double-blind, double-dummy, parallel-group OPERA I and OPERA II trials (OPERA I/NCT01247324 and OPERA II/NCT01412333), and in the phase III, randomized, placebo-controlled, double-blind, multicenter ORATORIO trial (NCT01194570). Compared to subjects with RMS, subjects with PPMS had slightly higher numbers of SELs (6.3 vs 4.6), and proportion of baseline T2-weighted lesion volume identified as SELs (11.3% vs 8.6%). The differences remained significant after adjusting for age, gender, and Expanded Disability Status Scale (EDSS) score (data on file) suggesting that SELs increase (but only slightly) as MS becomes more clinically progressive. When treatment effect was computed in ORATORIO patients, ocrelizumab showed an effect on reducing the overall accumulation of damage within pre-existing lesions, including SELs, but the effect was only modest.

The same methodology applied to an independent data set including subject with relapsing-remitting multiple sclerosis (RRMS) and SPMS^4^ identified more than 1 SEL in the majority of the patients in both groups (SPMS: 89%; RRMS: 83%). The SPMS population tended to have higher numbers and volume of SELs, but the differences were not significant. In all groups, tissue destruction, as evidenced by lower magnetisation transfer ratio, lower normalized T1 intensity and higher radial diffusivity, was greater in SELs than in the T2 lesion volume outside of SELs.^4^

Similar observations on SELs have been made by others in secondary progressive MS^5^ and in RRMS patients treated with fingolimod or natalizumab^6^ (taking into account the fact that the volume and number of SELs measured in different laboratories are not directly comparable due the use of different detection algorithms).

## The histopathology evidence for SELs being a marker of PMS

Chronic active lesions detected at post-mortem are most commonly found in progressive MS.^5^ However, they are also present in RMS,^7^ and estimates of the greater prevalence of chronic active lesions in PMS based on post-mortem studies have to be interpreted in the light of the observational bias associated with the fact that patients with RRMS usually develop PMS before they die. MRI provides a means for detecting SELs in vivo that avoids the bias associated with post-mortem studies. SELs show only partial correspondence with PRLs, suggesting that SELs with or without phase rims and PRLs with or without slow expansion represent different aspects or stages of MS pathology within chronic active lesions.^8^

## The MRI evidence for SELs being a marker of progression in both RMS as well as PMS

SELs have been shown to predict progression in PPMS,^9^ where it has long been known that increases in disability occur independent of relapse activity. More recently, it has become clear that the majority of the increase in disability in RMS also occurs independent of relapse activity;^10^ SEL volume was associated with this form of progression in ocrelizumab-treated RMS subjects in the Opera trials, and the T1 lesion volume associated with SELs at baseline predicted disease progression in the extension phase of these trials (data on file).

## Conclusion

Thus, MRI data do not support the proposition that SELs are a specific marker of PMS as traditionally defined clinically but rather indicate that they are a marker of progressive *biology*, which is increasingly appreciated to occur from the onset of MS throughout the disease course.
